# Unique Genetic and Histological Signatures of Mouse Pericardial Adipose Tissue

**DOI:** 10.3390/nu12061855

**Published:** 2020-06-22

**Authors:** A. Al-Dibouni, R. Gaspar, S. Ige, S. Boateng, F. R. Cagampang, J. Gibbins, R. D. Cox, D. Sellayah

**Affiliations:** 1 School of Biological Sciences, University of Reading, Whiteknights, Reading, Berkshire RG6 6AS, UK; alaa.al-dibouni@pgr.reading.ac.uk (A.A.-D.); r.simoesgaspar@pgr.reading.ac.uk (R.G.); Susan.Ige@pgr.reading.ac.uk (S.I.); s.boateng@reading.ac.uk (S.B.); j.m.gibbins@reading.ac.uk (J.G.); 2 Institute of Developmental Sciences, Human Development and Health, School of Medicine, University of Southampton, Southampton SO16 6YD, UK; f.cagampang@soton.ac.uk; 3 MRC Harwell Institute, Genetics of Type 2 Diabetes, Mammalian Genetics Unit, Harwell Campus, Oxfordshire OX11 0RD, UK; r.cox@har.mrc.ac.uk

**Keywords:** adipose tissue, obesity, metabolism

## Abstract

Obesity is a major risk factor for a plethora of metabolic disturbances including diabetes and cardiovascular disease. Accumulating evidence is showing that there is an adipose tissue depot-dependent relationship with obesity-induced metabolic dysfunction. While some adipose depots, such as subcutaneous fat, are generally metabolically innocuous, others such as visceral fat, are directly deleterious. A lesser known visceral adipose depot is the pericardial adipose tissue depot. We therefore set out to examine its transcriptional and morphological signature under chow and high-fat fed conditions, in comparison with other adipose depots, using a mouse model. Our results revealed that under chow conditions pericardial adipose tissue has uncoupling-protein 1 gene expression levels which are significantly higher than classical subcutaneous and visceral adipose depots. We also observed that under high-fat diet conditions, the pericardial adipose depot exhibits greatly upregulated transcript levels of inflammatory cytokines. Our results collectively indicate, for the first time, that the pericardial adipose tissue possesses a unique transcriptional and histological signature which has features of both a beige (brown fat-like) but also pro-inflammatory depot, such as visceral fat. This unique profile may be involved in metabolic dysfunction associated with obesity.

## 1. Introduction

Obesity is a major risk factor for a plethora of metabolic disturbances and diseases, including heart disease, which is a leading cause of death in most industrialised countries [1]. Ectopic fat deposition, which may refer to accumulation of triglycerides beyond the adipose tissue or the presence of non-classical adipose tissue depots is commonly associated with increased cardiovascular risk and poorer clinical outcomes [2,3], with a number of genetic determinants of ectopic fat having been identified [4].

The classical adipose tissue depots can be classified into two major types: white adipose tissue (WAT) and brown adipose tissue (BAT), the latter of which is concerned with thermogenesis and lipolysis, while the former is associated with lipid storage [5,6]. WAT is comprised of a range of depots, which are anatomically, genetically and functionally distinct [7,8]. In humans and animals, visceral WAT depots include intra-abdominal fat and gonadal fat, respectively. These depots are associated with metabolically deleterious hypertrophic adipose tissue growth and inflammation, whereas, subcutaneous WAT depots are associated with lower inflammatory profiles and reduced cardiometabolic risk [9,10].

**Figure 1:**
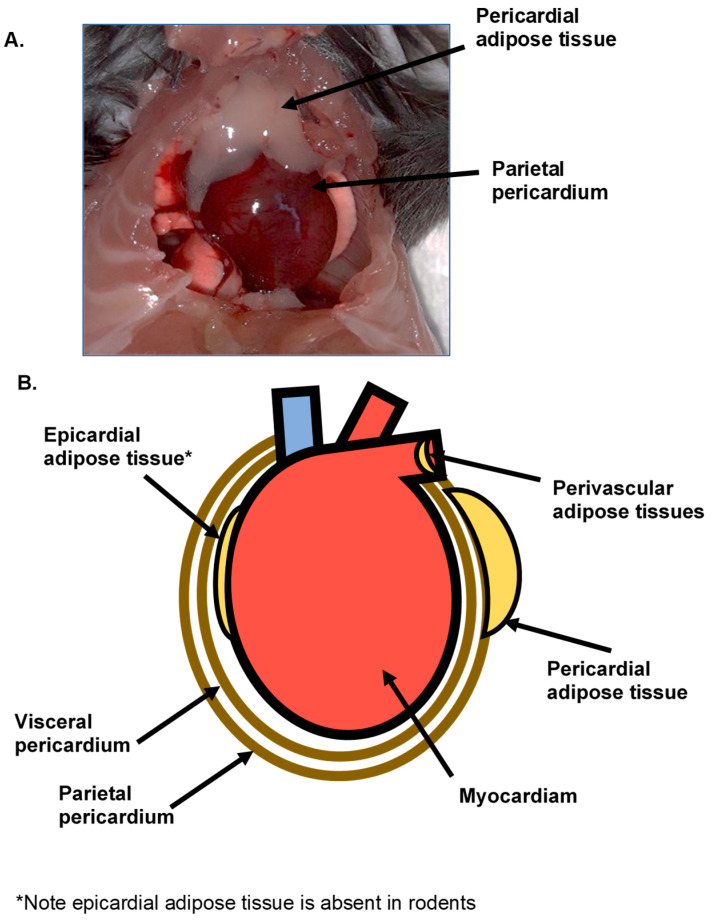
The location of epicardial adipose tissue (EAT), pericardial adipose tissue (PAT) and perivascular adipose tissue (PVAT) associated with the heart in rodents (**A**) and diagrammatically in humans (**B**). EAT is absent in the heart of rodents but present in the heart of human.

The human heart is in proximity to numerous adipose tissue depots, the nomenclature and classification of which has in the past been the subject of confusion [11,12]. These include epicardial adipose tissue (EAT), pericardial adipose tissue (PAT) and perivascular adipose tissue (PVAT) [11,13], as indicated in Figure 1. It is now established that while there may be an overlap in their embryonic origins, the ectopic fat depots within the vicinity of the heart are anatomically distinct and may have specific physiological relevance regarding the pathogenesis of obesity-associated cardiovascular disease [11,12,13]. While EAT is located between the myocardium and the inner layer of the pericardium, PAT is not in contact with the myocardium and is located anterior to the pericardial sac. (Figure 1B). The distinction between the epicardial and pericardial adipose depots was defined clearly in a recent review [14].

Human EAT, which can be observed through various imaging modalities, shares a vascular network with the myocardium and is thus uniquely positioned to impact cardiovascular risk [15,16]. As with classical visceral adipose tissue, both pericardial and epicardial depots in humans have been linked with increased inflammatory profiles and various measures of cardiometabolic risk, including vascular calcification and their expansion in obesity potentially play important roles in the pathophysiology of cardiovascular disease and clinical outcomes [17,18,19,20]. Interestingly, human EAT has been shown to have genetic and histological signatures that resemble a thermogenic or ‘beige’ adipose tissue depot [21]. Recent studies in mice have suggested that PAT has unique genetic signatures compared to other classical adipose depots, though such studies have not included detailed transcriptional and histological analyses under different dietary paradigms [22,23].

EAT is the most extensively studied cardiovascular adipose depot in humans but appears to be absent in rodents [23]. PAT is present in both rodents and humans, and its volume is positively correlated with obesity-related metabolic syndrome and cardiac abnormalities [17,23,24]. Given the importance of the mouse in modelling metabolic disease processes associated with obesity, surprisingly little is known about how mouse PAT compares to that of classical adipose tissues, transcriptionally and histologically. We therefore explored the transcriptional signatures of PAT, with a specific focus of inflammatory, thermogenic and adipogenic genes, to determine whether distinctive expression profiles in this adipose tissue underlie distinctive function and potential relationship to cardiovascular dysfunction. We also assessed the morphological characteristics of PAT through histological analysis.

Our results demonstrate that PAT depot has unique genetic and histological signatures, with thermogenic gene profiles that are intermediate between subcutaneous and brown fat depots, but also having an inflammatory transcriptional profile comparable to that of visceral adipose tissue. This study may have important experimental and clinically relevant implications that further our understanding of the role of pericardial adipose tissue in metabolic disease.

## 2. Methods

### 2.1. Animal and Experimental Design

All animal studies were approved by the Medical Research Council Harwell Institute Animal Welfare and Ethical Review Board, and all procedures were carried out within project license restrictions (PPL 30/3146) under the UK Animals (Scientific Procedures) Act 1986, issued by the UK Government Home Office. All mice were maintained on 12 hr light:dark cycle with ad libitum access to food. Four-week old male C57BL6/N mice were randomly assigned to either a chow (C, 10% kCal fat) or high fat (HF, 60% kCal fat) for 26 weeks. Throughout the dietary intervention, mice were weighed weekly, metabolic cage analysis performed at 22 weeks using TSE PhenoMaster in vivo calorimetry system, whilst body composition and intraperitoneal glucose tolerance test (ipGTT) were measured at 26 weeks of age. At 30 weeks of age, blood was collected by cardiac puncture under terminal anaesthetic. For serological analysis, blood was allowed to clot, then centrifuged at 3000 × g for 3 min and sera frozen at −80 °C until analysis. All samples for serum biochemistry analyses including cholesterol, fatty acids and triglycerides analysis were conducted using an AU680 Clinical Chemistry Analyser (Beckman Coulter, High Wycombe, UK), by the Clinical Chemistry core facility, MRC Harwell Institute (Oxfordshire, UK) using commercially-available kits and performed according to manufacturer instructions. Body composition was measured at 26 weeks of age by nuclear magnetic resonance (EchoMRI™, Zinsser Analytic GmbH, Eschborn, Germany) which determined total body fat, lean mass and free fluid in grams. The percentage of each component was then calculated based on the total body weight of the animal. Intraperitoneal glucose tolerance tests were performed at 26 weeks following an 8-h fast. Specifically, fasted mice received an i.p. administration of 2 g/kg glucose and blood sampled from the tail vein under a local anaesthetic (EMLA cream, AstraZeneca, Cambridge, UK) at 0 min (baseline), 15, 30, 60 and 120 min post glucose injection. Whole blood glucose was measured using an AlphaTRAK meter and test strips (Abbott Animal Health, Kent, UK).

### 2.2. RNA Isolation

One millilitre (1 mL) of Invitrogen™ TRI Reagent™ Solution per 50 mg to 100 mg of gonadal white adipose tissue (gWAT), inguinal white adipose tissue (iWAT), intercapsular brown adipose tissue (iBAT) and pericardial adipose tissue (PAT) was transferred to a MP Biomedicals^TM^ Lysing Matrix D tube. Lysing tubes were placed in the MP Biomedicals™ FastPrep-24™ 5G Instrument, with the QuickPrep Adaptor (Fisher Scientific) and homogenised at a speed of 6.0 m/s, for 40 s. Isolation of RNA was performed as recommended by the manufactures of Invitrogen™ TRI Reagent™ Solution (Fisher Scientific).

### 2.3. cDNA Synthesis and qRT-PCR

Total RNA was reverse transcribed using the Applied Biosystems™ High-Capacity cDNA Reverse Transcription Kit (Fisher Scientific) and Invitrogen™ RNaseOUT™ Recombinant Ribonuclease Inhibitor (Fisher Scientific) following the instructions as recommended by the manufacturer to synthesise cDNA, performed using the T100™ Thermal cycler (Bio-Rad). The gene expression of 30 ng cDNA was determined using qPCRBIO Probe Mix No-ROX (PCR Biosystems), performed on the MyiQ™ Single-Colour Real-Time PCR Detection System (Bio-Rad). Samples were measured in duplicates and the fold change in gene expression levels were determined using the comparative threshold cycle (CT) method, also referred to as the 2^−^^ΔΔ*C*t^ method. The target genes listed below were normalized to the housekeeping gene, peptidylprolyl isomerase A (PPIA). All Applied Biosystems™ TaqMan™ Gene Expression Assay (FAM-MGB) (Table 1) were purchased from Fisher Scientific.

### 2.4. Histology

Formalin fixed gWAT, iWAT and PAT embedded in Optimal Cutting Temperature (OCT) compound were sectioned between 10 µm and 15 µm using a Bright 5040 Cryostat (Bright Instruments Limited). Sections were stained with Haematoxylin and Eosin (H&E) and imaged with a Nikon TE200 Brightfield Inverted Microscope at 40× objective lens. Assuming the shape of adipocytes are spherical, to calculate adipocyte volume, Image-J software was used to determine adipocyte area, as stated below. The percentage change in volume was determined by normalising adipocyte volume to the chow-fed mice, unless stated otherwise, and multiplying by 100.

To determine adipocyte volume from adipocyte area:

Radius = √(Area/π)

Radius^3^ = Radius^3

Volume of a sphere = (4/3)∗(π∗Radius^3^)

To assess the adipocyte size distribution pattern the raw values of the adipocyte, volumes derived from image-J were used to determine the frequency distribution of cells. This was achieved by using the frequency function as an array formula in excel and calculating the percentage of cells present within each cell volume category (binning) across the adipocyte size spectrum (presented as arbitrary units).

### 2.5. Statistical Analysis

GraphPad Prism 8 was used to perform statistical analysis of one-way ANOVA (with post-hoc Tukey’s comparison), unpaired *t*-test or Pearson’s correlation coefficient. All values are expressed as mean + standard error of the mean (s.e.m). A value of *p* < 0.05 was deemed as statistically significant with * *p* < 0.05, ** *p* < 0.01, *** *p* < 0.001 and **** *p* < 0.0001.

## 3. Results

### 3.1. Adipose Tissue Gene Expression Differences at Baseline in Chow-Fed Mice

**Figure 2:**
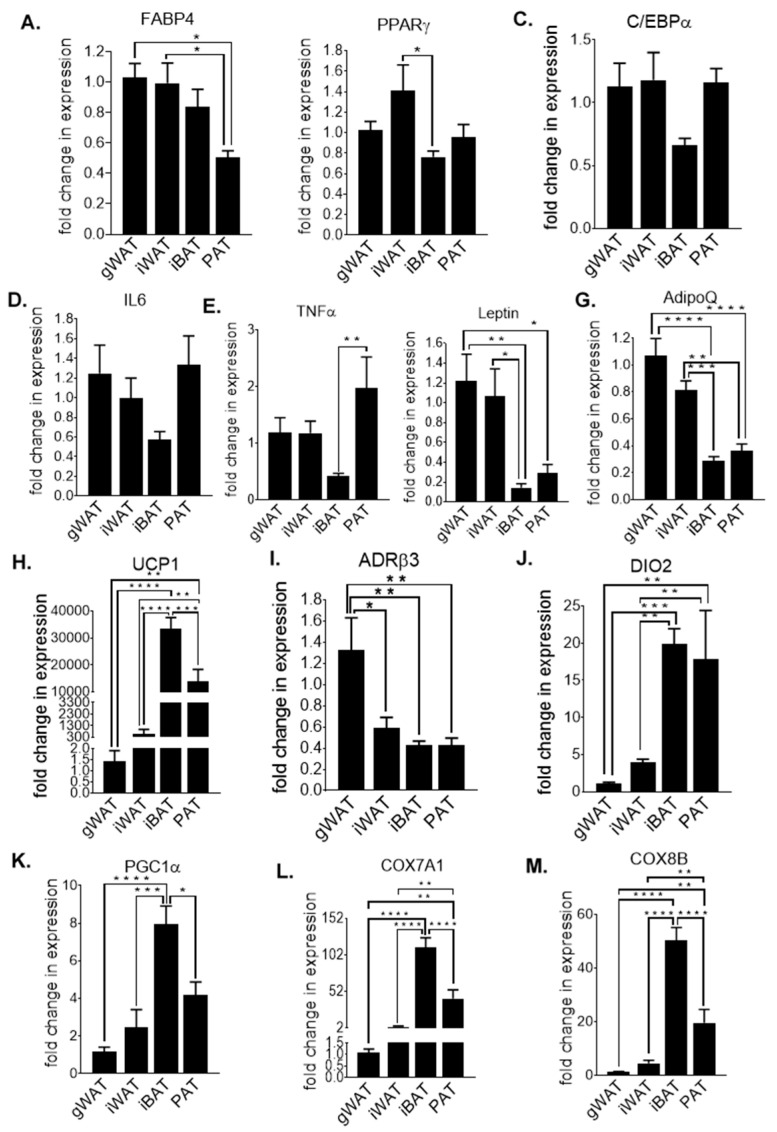
FABP4 (**A**), PPARγ (**B**), C/EBPα (**C**), IL6 (**D**), TNFα (**E**), Leptin (**F**), AdipoQ (**G**), UCP1 (**H**), ADRβ3 (**I**), DIO2 (**J**), PGC1α (**K**), COX7A1 (**L**) and COX8B (**M**) fold change in mRNA expression in gWAT, iWAT, iBAT and PAT of chow (C)-fed 30-week-old mice. All data are relative to the housekeeping gene PPIA and normalised to C-gWAT. All data represent mean + s.e.m. Data were analysed by one-way ANOVA and Tukey’s multiple comparison test. N = 8–12. * *p* < 0.05, ** *p* < 0.01, *** *p* < 0.001 and **** *p* < 0.0001.

For comparison of the transcriptional alterations occurring at baseline, markers associated with adipogenesis (Figure 2A–C), inflammation (Figure 2D–G), thermogenesis (Figure 2H–J) and mitochondrial function (Figure 2K–M) were assessed in gWAT, iWAT, iBAT and PAT, in 30-week-old male mice fed a standard chow diet. The gene expression of fatty acid-binding protein 4 (FABP4) (Figure 2A), an adipocyte terminal differentiation marker, was significantly lower, with a ~50% decrease in PAT compared to iWAT (*p* < 0.05) and gWAT (*p* < 0.05). In addition, the ‘master regulators of adipogenesis’, peroxisome proliferator-activated receptor gamma (PPARγ) (Figure 2B) and CCAAT/enhancer binding protein (C/EBPα) (Figure 2C), exhibited a similar transcriptional profile, but only iBAT showed a significant reduction in PPARγ gene expression compared to iWAT (*p* < 0.05), with no other significant changes between the adipose tissue depots in these genes. In this case, PAT from chow-fed mice resembles a similar gene expression level of pro-adipogenic markers as gWAT, at baseline.

The response to pro-inflammatory, depot-specific markers was assessed by observing the changes in the transcript levels of interleukin 6 (IL6) (Figure 2D) and tumour necrosis factor alpha (TNFα) (Figure 2E). A significantly higher expression of the pro-inflammatory cytokine TNFα in PAT compared to BAT (*p* < 0.01) was observed. Although there were no significant changes in IL6 expression, iBAT IL6 expression was >40% lower compared to gWAT, iWAT and PAT.

As well as IL6 and TNFα, adipocytes also secrete pro-inflammatory adipokines, such as leptin (Figure 2F), and anti-inflammatory adipokines, such as adiponectin (AdipoQ) (Figure 2G). The transcription levels between the four adipose tissue depots illustrate a similar profile in both leptin and AdipoQ, with gWAT exhibiting the highest levels of expression (1.22 and 1.07, respectively) followed by iWAT (1.07 and 0.81, respectively), PAT (0.30 and 0.36, respectively) and iBAT (0.14 and 0.29, respectively). A significantly lower expression of leptin in iBAT compared to gWAT (*p* < 0.01) and iWAT (*p* < 0.05) was observed. There was also a significantly lower expression of leptin in PAT compared to gWAT (*p* < 0.05) and a strong trend towards a lower expression level of leptin in PAT compared to iWAT (*p* = 0.0771). AdipoQ levels in iBAT and PAT displayed lower expression compared with iWAT (*p* < 0.001 and *p* < 0.01, respectively) and gWAT (*p* < 0.0001 and *p* < 0.0001, respectively).

Brown adipose tissue specific markers such as uncoupling protein 1 (UCP1) (Figure 2H), beta-3 adrenergic receptor (ADRβ3) (Figure 2I) and deiodinase 2 (DIO2) (Figure 2J), play a role in the thermogenic and lipolytic properties of the tissue. UCP1 levels were significantly higher in iBAT than those of gWAT (*p* < 0.0001) by >20,000-fold increase and iWAT with >50-fold increase (*p* < 0.0001) and >2-fold higher than that of PAT (*p* < 0.001). In fact, PAT UCP1 levels were >9000 fold higher than gWAT (*p* < 0.01) and >20-fold higher than iWAT (*p* < 0.01). On the contrary, ADRβ3 levels were highest in gWAT (1.32) compared to 0.59 in iWAT (*p* < 0.05), 0.43 in iBAT (*p* < 0.01) and 0.43 in PAT (*p* < 0.01), with no significant differences amongst other depots. Similar to UCP1, DIO2 showed a similar profile as UCP1, in which expression was significantly higher in iBAT (19.86) and PAT (17.80) compared to a transcript level of 3.92 in iWAT (*p* < 0.01 and *p* < 0.01, respectively) and a transcript level of 1.10 in gWAT (*p* < 0.001 and *p* < 0.01), respectively).

Peroxisome proliferator-activated receptor gamma coactivator 1-alpha (PGC1α) (Figure 2K) and cytochrome c oxidase subunit 7A1 (COX7A1) (Figure 2L) and 8B (COX8B) (Figure 2M) are well known markers to determine mitochondrial function. As with UCP1 transcript levels, PGC1α, COX7A1 and COX8B levels were highest in iBAT (7.93, 112.4 and 50.41, respectively), intermediate in PAT (4.17, 42.31 and 19.50, respectively) and lowest in iWAT (2.45, 3.34 and 4.39, respectively) and gWAT (1.18, 1.09 and 1.15, respectively). For PGC1α, statistically significant differences were observed in iBAT compared to iWAT (*p* < 0.001), gWAT (*p* < 0.0001) and PAT (*p* < 0.05). While iBAT COX7A1 and COX8B expression were significantly higher than those of gWAT (*p* <0.0001 and *p* < 0.0001, respectively), iWAT (*p* < 0.0001 and *p* < 0.0001, respectively) and PAT (*p* < 0.0001 and *p* < 0.0001, respectively), expression levels were significantly higher in PAT compared to gWAT (*p* <0.01 and *p* <0.01, respectively) and iWAT (*p* <0.01 and *p* <0.01, respectively). As expected, transcription levels of brown adipocyte-associated markers like UCP1, which is highly expressed in brown fat, were extremely high compared to gWAT and iWAT. However, although PAT UCP1 expression was lower compared to iBAT, this was to a much lower extent, suggesting that PAT from chow-fed mice may possess thermogenic properties that have not been recognized before.

### 3.2. Adipose Tissue Gene Expression Differences in Chow-Fed Mice and HF-Fed Mice

In order to compare the adipogenic response of different adipose depots in obesity, mice were fed a high-fat (HF) diet for 26 weeks and compared to chow (C)-fed counterparts. Metabolic and phenotypic data supporting obesity and other metabolic dysfunctions in HF-fed animals are presented in Supplementary Figure S1. The adipogenic gene expression levels were also assessed in these 30-week-old male HF-fed animals (Supplementary Figure S2A–C) and compared to their respective chow-fed counterparts.

There were no statistically significant changes in HF-fed mice compared to chow-fed mice in gWAT, iWAT, iBAT or PAT in FABP4 expression (Supplementary Figure S2A). However, HF-fed mice exhibited significantly lower expression levels of PPARγ in gWAT (*p* < 0.01) and in PAT (*p* < 0.05), and a trend towards significance in iBAT (*p* = 0.054), compared to their chow fed-counterparts (Supplementary Figure S2B). We observed significantly higher expression of C/EBPα in iBAT (*p* < 0.05) and a trend towards lower expression in gWAT (*p* = 0.0616) and PAT (*p* = 0.0515), but no significant differences in iWAT. PAT HF-fed mice have the lowest gene expression profile in FABP4 (0.71), PPARγ (0.55) and C/EBPα (0.65) compared to gWAT (0.97, 0.62 and 0.68, respectively), iWAT (1.10, 0.70 and 1.13, respectively) and iBAT (1.03, 1.30 and 1.75, respectively) between all adipogenic markers, suggesting a lower adipogenic potential in PAT HF-fed mice versus PAT chow-fed mice, compared to the other depots.

**Figure 3:**
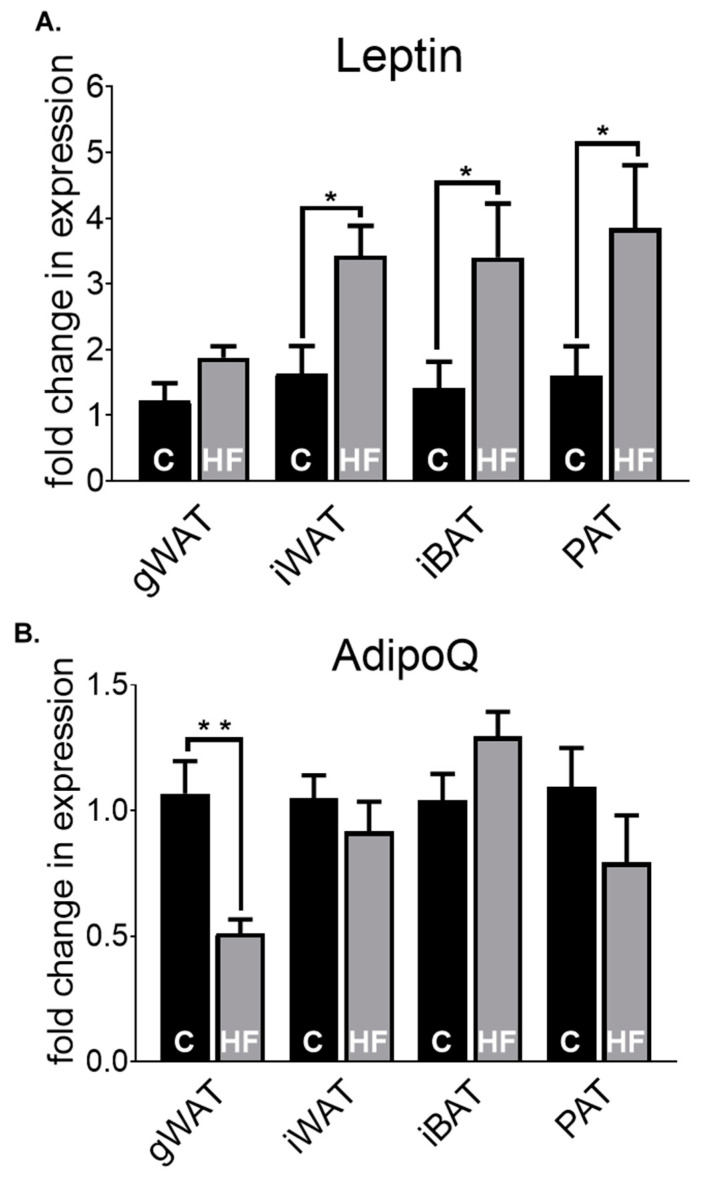
Leptin (**A**) and AdipoQ (**B**) fold change in mRNA expression in gWAT, iWAT, iBAT and PAT of chow (**C**)- or high-fat (HF)-fed 30-week-old male mice. All data are relative to the housekeeping gene PPIA and normalised to their respective control (C) within each depot. All data represent mean + s.e.m. Data were analysed by an unpaired *t*-test between C and HF of each depot. N = 6–12. * *p* < 0.05 and ** *p* < 0.01.

As shown in Figure 2, we assessed the profiles of a range of genes in chow, but how will the transcripts of key adipokines differ in the adipose depots induced by a HF-diet? We specifically analysed leptin (Figure 3A) and AdipoQ expression (Figure 3B) in the gWAT, iWAT, iBAT and PAT in 30-week-old chow (C) and high-fat (HF)-fed mice. There were significantly higher gene expression levels of leptin in HF-fed mice compared to C-fed counterparts in iWAT (*p* < 0.05), iBAT (*p* < 0.05) and PAT (*p* < 0.05). Leptin in PAT HF-fed mice have a higher transcript level (3.85), followed by iWAT (3.43), iBAT (3.40) and gWAT (1.88) when compared to their respective chow groups. AdipoQ expression was significantly lower in gWAT HF-fed mice (*p* < 0.01) compared to gWAT chow-fed mice, but no other significant changes were observed within the other depots. In general, PAT shows a similar expression pattern in both leptin and AdipoQ expression to that of gWAT and iWAT, with HF-fed animals having higher expression in leptin and lower expression in AdipoQ compared to their chow-fed counterparts.

**Figure 4:**
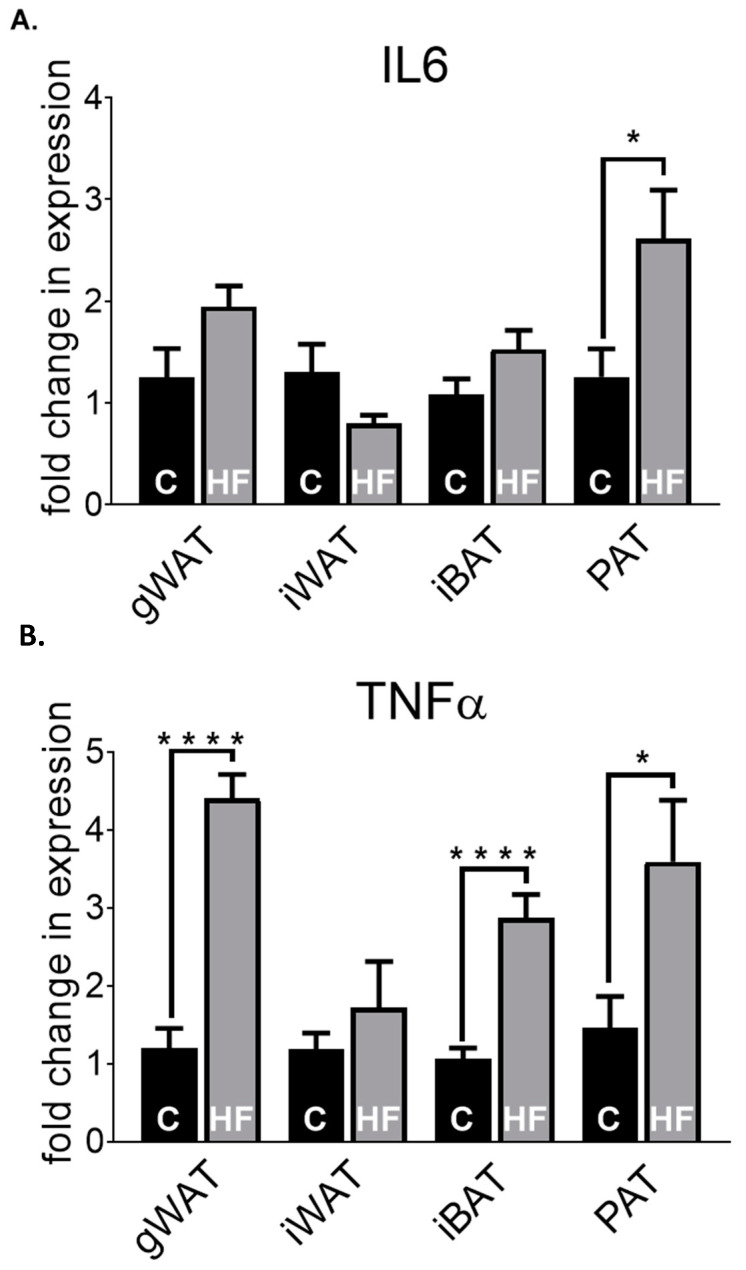
IL6 (**A**) and TNFα (**B**) fold change in mRNA expression in gWAT, iWAT, iBAT and PAT of chow (C)- or high-fat (HF)-fed 30-week-old male mice. All data are relative to the housekeeping gene PPIA and normalised to their respective control (C) within each depot. All data represent mean + s.e.m. Data were analysed by an unpaired *t*-test between C and HF of each depot. N = 6–12. * *p* < 0.05 and **** *p* < 0.0001.

As obesity, induced by a state of high caloric intake, is associated with chronic inflammation, we next examined whether the pro-inflammatory cytokines IL6 (Figure 4A) and TNFα levels (Figure 4B) were altered in by HF diet in gWAT, iWAT, iBAT and PAT compared to their chow-fed counterparts. We observed >2-fold increase in IL6 levels in PAT of HF-fed mice compared to that of chow-fed mice (*p* < 0.05), with no other significant changes occurring between the other depots. Expression levels of TNFα were significantly higher in gWAT (*p* <0.0001), iBAT (*p* <0.0001) and PAT (*p* < 0.05) of HF-fed mice compared to chow-fed mice, but not in iWAT. In PAT, there was a significantly higher expression of both IL6 and TNFα in HF-fed mice compared to chow-fed mice. Indeed, PAT was the only depot wherein both pro-inflammatory markers were upregulated by HF-diet.

**Figure 5:**
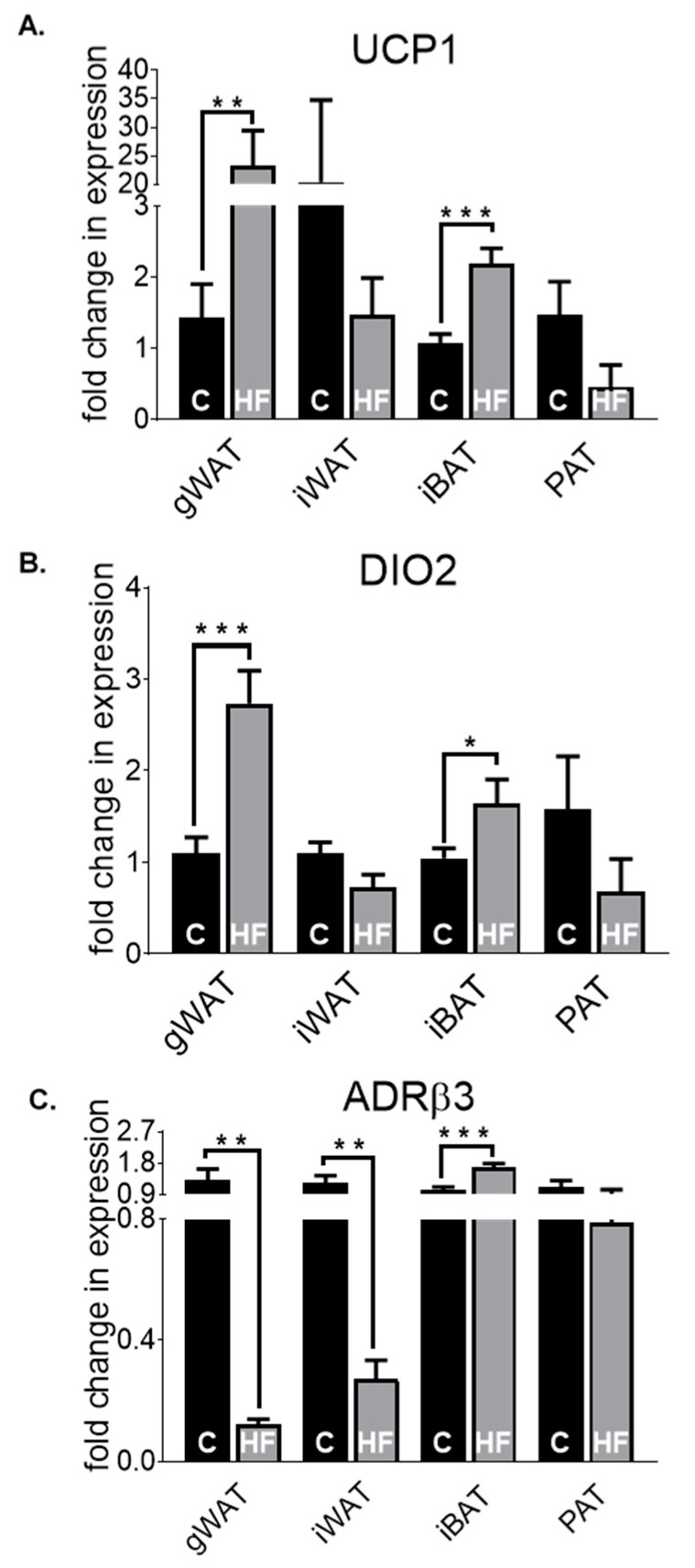
UCP1 (**A**), ADRβ3 (**B**) and DIO2 (**C**) fold change in mRNA expression in gWAT, iWAT, iBAT and PAT of chow (C)- or high-fat (HF)-fed 30-week-old male mice. All data are relative to the housekeeping gene PPIA and normalised to their respective control (C) within each depot. All data represent mean + s.e.m. Data were analysed by an unpaired *t*-test between C and HF of each depot. N = 6–12. * *p* < 0.05, ** *p* < 0.01 and *** *p* < 0.001.

Next, the thermogenic properties in gWAT, iWAT, iBAT and PAT between chow- and HF-fed mice were evaluated. UCP1 (Figure 5A) levels were significantly higher in gWAT (*p* < 0.01) and iBAT (*p* < 0.001) in HF-fed mice, normalised to chow-gWAT and chow-iBAT, respectively, with no significant changes displayed in iWAT or PAT. A similar transcript profile was seen in DIO2 (Figure 5B) as UCP1, in gWAT (*p* < 0.001), iWAT, iBAT (*p* < 0.05) and PAT. ADRβ3 (Figure 5C) gene expression was significantly lower in HF-fed mice in gWAT (*p* < 0.01) and iWAT (*p* < 0.01), but higher in iBAT (*p* < 0.001), with no differences exhibited in PAT. Generally, PAT expression in thermogenic markers mirrors that of iWAT, with a tendency to lower gene expression levels in HF-fed mice compared to its chow-fed counterpart. The expression signatures of mitochondrial function markers (Supplementary Figure S3A–C) in gWAT, iWAT, iBAT and PAT showed no statistically significant differences between chow- and HF-fed mice.

### 3.3. Adipose Tissue Gene Expression Differences in HF-Fed Mice

**Figure 6:**
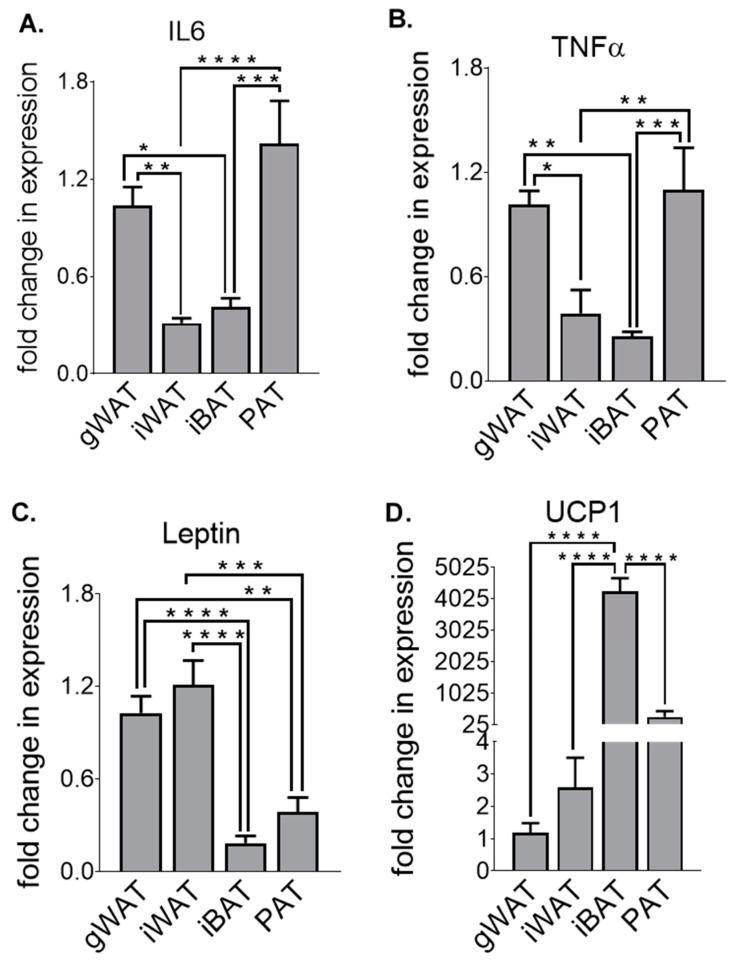
IL6 (**A**), TNFα (**B**), Leptin (**C**) and UCP1 (**D**) fold change in mRNA expression in gWAT, iWAT, iBAT and PAT of high-fat (HF)-fed 30-week-old male mice. All data are relative to the housekeeping gene PPIA and normalised to HF-gWAT. All data represent mean + s.e.m. Data were analysed by one-way ANOVA and Tukey’s multiple comparison test. N = 6–8. * *p* < 0.05, ** *p* < 0.01, *** *p* < 0.001 and **** *p* < 0.0001.

Similarly to Figure 2, we then determined the depot-specific transcriptional profile of inflammatory (Figure 6A,B), adipokine (Figure 6C) and thermogenic (Figure 6D) transcriptional profiles in HF-fed of 30-week-old male mice, using gWAT HF-fed mice as the control for comparison. We observed significantly higher expression of the pro-inflammatory cytokines IL6 and TNFα in PAT compared to iBAT (*p* < 0.001 and *p* < 0.001, respectively) and iWAT (*p* < 0.0001 and *p* < 0.01, respectively). In addition, there was significantly lower expression of IL6 and TNFα in iWAT (*p* < 0.01 and *p* < 0.05, respectively) and iBAT (*p* < 0.05 and *p* < 0.01 respectively) compared to gWAT. Leptin levels were significantly lower in PAT and iBAT compared to gWAT (*p* < 0.01 and *p* < 0.0001, respectively) and iWAT (*p* < 0.001 and *p* < 0.0001, respectively). UCP1 levels were highest in iBAT (4251.00), intermediate in PAT (268.90) and lowest in iWAT (2.58) and gWAT (1.18). Statistically significant differences were observed in iBAT compared to gWAT (*p* <0.0001), iWAT (*p* < 0.0001) and PAT (*p* < 0.0001).

### 3.4. Adipose Tissue Histological Differences in Chow-Fed and HF-Fed Mice

**Figure 7:**
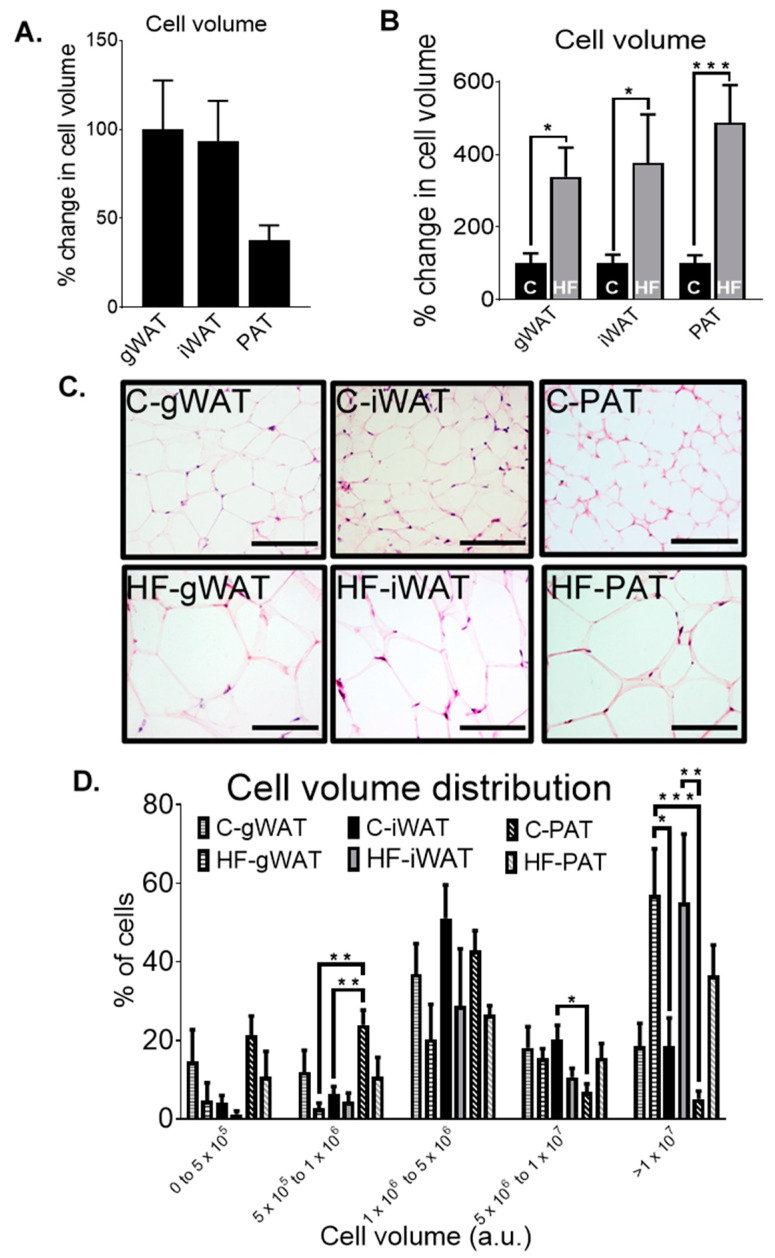
Percentage change in adipocyte volume (a.u.) of gWAT, iWAT and PAT from 30-week-old chow (**C**)-fed mice. Data normalised to C-gWAT. Data were analysed by one-way ANOVA and Tukey’s multiple comparison test. N = 8–11 (**A**). Percentage change in adipocyte volume of chow (C)- or high-fat (HF)-fed 30-week-old mice from gWAT, iWAT and PAT. Data normalised to their respective control (**C**) within each depot. Data were analysed by an unpaired *t*-test between C and HF of each depot. N = 6–11 (B). Representative H&E stained images of chow (C)- or high-fat (HF)-fed 30-week-old mice from gWAT, iWAT and PAT. All scale bars 100µM. N = 6–11 (**C**). Distribution of adipocyte volume of chow (C)- or high-fat (HF)-fed 30-week-old mice from gWAT, iWAT and PAT. Data were analysed by one-way ANOVA and Tukey’s multiple comparison test between gWAT, iWAT and PAT of each cell volume (a.u.) bin. N = 6-11 (D). All data represent mean + s.e.m (**A**,**B**,**D**). * *p* < 0.05, ** *p* < 0.01 and *** *p* < 0.001 (**A**,**B**,**D**).

To determine the differences in adipocyte volume at baseline of gWAT, iWAT and PAT (Figure 7A), we conducted histological analysis of these depots at 30 weeks old in chow-fed mice. We observed no differences in adipocyte volume between gWAT, iWAT or PAT. However, PAT had ~60% reduction in cell volume compared to the other depots, and a trend towards a smaller depot volume compared to gWAT (*p* = 0.078), with morphologically smaller cells compared to gWAT and iWAT (Figure 7C).

Next, we assessed the response of a HF-diet on adipocyte volume compared to a standard chow diet, histologically, in gWAT, iWAT, and PAT (Figure 7B,C). HF-fed mice had a significantly larger adipocyte volume in gWAT (*p* < 0.05), iWAT (*p* < 0.05) and PAT (*p* < 0.001) compared to their chow-fed counterparts. PAT displayed the largest volume in HF-fed mice (487.60%), followed by iWAT (376.80%) and gWAT (338.40%) compared to their respective chow controls (100%) (Figure 7B). In addition, the increase in adipocyte volume was also observed morphologically, in which HF-fed mice in all the adipose tissue depots had visibly larger cells compared to their respective chow controls (Figure 7C).

To investigate whether PAT had altered adipocyte cell size distribution with regard to classical adipose depots, we assessed the % of cells in each size category ranging from smallest to largest size in gWAT, iWAT and PAT, from 30-week-old chow (C)- and high-fat (HF)-fed mice (Figure 7D). While there were no significant differences in the % of cells in the smallest size category across the depots, we did observe that there was a higher percentage of smaller cells in the second smallest size category, indicating that PAT has higher prevalence of smaller cells compared to iWAT (5 × 10^5^ to 1 × 10^6^ cell volume (a.u.) bin). Within the 5 × 10^6^ to 1 × 10^7^ cell volume (a.u.) bin, C-PAT had significantly fewer large adipocytes compared to C-iWAT (*p* < 0.05). Furthermore, within the bin containing the largest cell size (>1 × 10^7^ cell volume (a.u.) bin), C-PAT had significantly fewer larger cells compared to HF-gWAT (*p* < 0.001) and HF-iWAT (*p* < 0.01) and C-iWAT had significantly less larger cells compared to HF-gWAT (*p* < 0.05).

### 3.5. The Relationship between Biochemical and Metabolic Parameters, Gene Expression Levels and Cell Volume in Adipose Tissue in Chow- and HF-Fed Mice

From the collected experimental data, we investigated whether there was a relationship between the percentage change in adipocyte volume compared to percentage body fat in both chow- and HF-fed mice (Supplementary Figure S4). In gWAT (Supplementary Figure S4A) and PAT (Supplementary Figure S4C), there was a strong positive linear correlation between cell volume and percentage body fat (*p* < 0.05 and *p* < 0.001, respectively), whereas in iWAT (Supplementary Figure S4B), there was no correlation.

As metabolic disorders such as cardiovascular disease and obesity are associated with high levels of inflammatory molecules and elevated levels of cholesterol, we sought to determine the relationship between the gene expression levels of the cytokines IL6 and TNFα in different adipose depots and the serum total cholesterol levels of chow (C)- and high-fat (HF)-fed mice compared to the percentage change in adipocyte volume in gWAT (Supplementary Figure S5A–C), iWAT (Supplementary Figure S5D–F) and PAT (Supplementary Figure S5G–I). In gWAT and PAT, there was a strong positive linear correlation between IL6 (*p* < 0.01 and *p* < 0.01, respectively), TNFα (*p* < 0.01 and *p* < 0.05, respectively) and serum total cholesterol levels (*p* < 0.01, and *p* < 0.01, respectively), compared to the percentage change in cell volume. In iWAT, there were no changes between IL6, TNFα, serum total cholesterol and cell volume.

As the level of inflammation increases, the state of obesity and therefore percentage body fat also progresses; thus, we evaluated the relationship between the gene expression levels of IL6 and TNFα against the percentage body fat of chow (C)- and high-fat (HF)-fed mice in gWAT (Supplementary Figure S6A,B), iWAT (Supplementary Figure S6C,D), iBAT (Supplementary Figure S6E–F) and PAT (Supplementary Figure S5G–H). In gWAT and PAT, there was a strong positive linear correlation between IL6 (*p* < 0.05 and *p* < 0.01, respectively) and TNFα expression (*p* <0.0001, and *p* < 0.05, respectively) compared to percentage body fat. In contrast to this, there was a strong negative linear correlation in IL6 expression (*p* < 0.001), but no correlation in TNFα expression compared to percentage body fat in iWAT. iBAT displayed a strong positive linear correlation in TNFα expression (*p* < 0.001), but no correlation between IL6 and percentage body fat. In all cases seen in Supplementary Figures S4–S6, the relationship between the independent variables in PAT resembles a similar correlation pattern as demonstrated in gWAT.

## 4. Discussion

Our data show that in 30-week-old male mice fed a chow diet, PAT UCP1 mRNA expression is >9000-fold higher than that of gWAT. While as expected, iBAT had the highest UCP1 expression (33462) of all the depots examined, PAT exhibited the second highest levels (13916). UCP1 is a hallmark of brown adipocytes and is responsible for the thermogenic properties of BAT [25]. BAT thermogenesis is energetically wasteful as UCP1 uncouples ATP synthesis from mitochondrial oxidative phosphorylation to liberate heat [26]. Since the ‘rediscovery’ of functionally active BAT in humans around 10 years ago, much attention has been given to the potential therapeutic relevance of BAT to combat obesity [27,28,29]. Certainly, BAT dysfunction has been linked to obesity and cardiovascular disease [27,28]. Recent evidence, however, suggests that adult human BAT might not reside in dedicated depots but instead resembles pockets of thermogenic brown adipocytes within white adipose tissue which may be recruited or expanded by adrenergic stimulation or exposure to cold [30]. Such white adipose depots that have the inherent capacity for thermogenic recruitment are referred to as beige adipose tissues and tend to have higher mRNA expression levels of UCP1 [31]. Rodent studies have shown that iWAT, which is the largest subcutaneous adipose tissue, represents a beige adipose depot wherein cold exposure leads to greatly induced UCP1 expression [32].

Our results show that PAT (13916) has considerably higher UCP1 expression levels than that of iWAT (578), suggesting the PAT in mice may resemble a beige depot that contains thermogenic active adipocytes at baseline. Similarly, to UCP1 expression, PAT had significantly higher mRNA expression levels of the thermogenic marker DIO2 compared to both gWAT and iWAT. DIO2 codes for an enzyme that catalyses the conversion 3,5,3′,5′-tetraiodothyronine (T4) to bioactive 3′,3′,5′-triiodothyronine (T3). Increased DIO2 levels are associated with increased BAT function and enhanced cold-induced adrenergic stimulation and thermogenesis. Mice with targeted disruption of the DIO2 gene have a failure in BAT lipid formation and mitochondrial respiration, even with compensatory upregulation of UCP1 [33]. PAT DIO2 mRNA levels at baseline were comparable to that of BAT and indicate the potential for high thermogenic capacity of PAT. Interestingly, the transcript levels of mitochondrial respiratory chain genes COX7A1 and COX8B was highest in iBAT and intermediate in PAT with the latter exhibiting significantly higher expression levels compared to gWAT and iWAT. A similar pattern of expression was observed for PGC1α, although did not reach statistical significance in PAT compared to gWAT and iWAT. Nevertheless, these data strongly suggest that under basal conditions PAT may represent a beige depot with a high thermogenic capacity. Indeed, further functional experiments are needed to explore this concept in detail.

While iBAT thermogenesis can be induced by cold or pharmacological beta-adrenergic stimulation, it can also be activated by high-fat diet. This phenomenon, known as diet-induced thermogenesis has been observed in numerous rodent studies and is driven by an upregulation and/or activation of UCP1 [34,35,36,37]. Our results showed that UCP1, DIO2 and ADRβ3 mRNA levels were induced by a 26-week high-fat diet in iBAT. Interestingly, gWAT UCP1 and DIO2 mRNA levels were also induced by a high-fat diet, although there was a significant downregulation in ADRβ3 mRNA levels in gWAT. The functional relevance of this surprising induction in gWAT UCP1 mRNA levels is unknown, and it may be that downregulation of ADRβ3 which is upstream of UCP1 in the thermogenic process in adipocytes counteracts the UCP1 induction. Indeed, increased thyroid hormone signalling through elevated DIO2 gene expression has been observed in obese subjects and may resemble thermoregulatory disturbances [38]. Moreover, in spite of the increased gWAT UCP1 expression levels under high-fat diet conditions, iBAT UCP1 levels remained considerably higher than that of gWAT. PAT transcript levels for these thermogenic markers showed reductions in the high-fat group compared to the chow-fed group, although this did not reach statistical significance. Human epicardial adipose tissue, which is anatomically distinct from pericardial adipose tissue, also appears to have high thermogenic genetic signatures, though its function in relation to cardiovascular physiology remains to be established [21].

Adipokines have been shown to have a major impact on metabolic function through a series of autocrine, paracrine and endocrine actions [39,40]. One of the key adipokines associated with metabolic health is leptin, which is produced by adipocytes and functions in body weight homeostasis. Our results show that, of all the depots examined in this study, iBAT and PAT showed the lowest levels of leptin mRNA expression under chow conditions. While it has been established that brown adipocytes produce and secrete less leptin than white adipocytes, the finding that BAT and PAT have comparable levels of leptin is further indication that PAT may resemble a beige depot as baseline [41,42]. Leptin mRNA levels are generally correlated with leptin secretion; both are related to adipocyte size and lipid droplet morphology and are under reciprocal regulatory control with that of UCP1 mRNA levels [43,44]. While higher leptin expression is associated with unilocularity and increased adipocyte size, lower leptin expression is associated with reduced adipocyte size and multilocularity which are hallmarks of brown adipocyte morphology [44]. Indeed, our histological analysis showed a very strong trend towards reduced adipocyte size in PAT compared to both gWAT and iWAT in chow fed mice. Undoubtedly, more direct analysis of beige characteristics in PAT, including UCP1 immunostaining are required to confirm this characterisation. Moreover, given that cold-induced upregulation of UCP1 mRNA expression in BAT and iWAT has been observed, it would be of interest to assess if similar interventions can induce PAT UCP1 expression [33].

In a state of high-fat-induced obesity, adipocytes increase their lipid storage capacity, resulting in hypertrophic growth. We see evidence of this hypertrophic adipocyte growth in all the depots examined though the increase from chow to high-fat feeding was most pronounced in PAT which exhibited a greater than four-fold increase in adipocyte size. Obesogenic hypertrophic adipocyte growth is associated with pro-inflammatory responses that promote insulin resistance and cardiovascular disease [40,45]. We observed that, of all the depots examined, only the PAT showed a significant increase in the mRNA expression of the two key pro-inflammatory cytokines in mouse adipose tissue, namely IL6 and TNFα. These results suggest that the high-fat diet may be potentiating pro-inflammatory macrophage infiltration and subsequent metabolic dysfunction. Indeed, human cardiac adipose depots such as pericardial and epicardial adipose tissue have been shown to have a high pro-inflammatory status that may be involved in the pathogenesis of coronary artery disease [39,46]. The visceral adipose depots in both rodent and human studies have been well documented to be more pro-inflammatory than subcutaneous depots and explain why visceral adipose depots are more closely associated with metabolic disease [45,47]. This depot-specific inflammatory response maybe due, at least in part, to the observation that visceral adipose tissue expands by hypertrophy whereas subcutaneous depots tend to expand by hyperplastic growth, which is a more healthy form of obesogenic expansion [45]. To this end, metabolically healthy obesity is associated with subcutaneous adipose expansion versus that of visceral [47,48]. To this end, our correlation analysis revealed a strong positive association between pro-inflammatory cytokine mRNA expression and adipocyte size in PAT and gWAT but not iWAT. We also observed a strong positive correlation between pro-inflammatory cytokine mRNA expression and both body fat % and serum cholesterol in PAT and gWAT but not iWAT. In fact, iWAT showed the opposite trend. Given that dense epicardial adipose tissue macrophage infiltrate in humans has been shown to be involved in the pathogenesis of necrotic plaque accretion in the vasculature, our results potentially point to a similar role for mouse pericardial adipose tissue.

Taken together, our results point to both a thermogenic and pro-inflammatory profile of mouse pericardial adipose tissue which represents a unique and previously unreported finding which is of potential and experimental clinical importance. Our results suggest that mouse PAT may have functional qualities that resemble both BAT as well as visceral adipose tissue. While further functional studies are required to confirm the physiological relevance of the genomic and histological analyses observed here, our results provide compelling evidence that mouse pericardial adipose tissue deserves closer attention in metabolic research.

## Figures and Tables

**Table 1:** List of TaqMan™ Gene Expression Assays

Gene	Assay ID
AdipoQ	Mm00456425_m1
ADRβ3	Mm02601819_g1
C/EBPα	Mm00514283_s1
COX7A1	Mm00438297_g1
COX8B	Mm00432648_m1
DIO2	Mm00515664_m1
FABP4	Mm00445878_m1
IL6	Mm00446190_m1
Leptin	Mm00434759_m1
PGC1α	Mm01208835_m1
PPARγ	Mm00440940_m1
PPIA	Mm02342430_g1
TNFα	Mm00443258_m1
UCP1	Mm01244861_m1

## Supplementary Materials

The following are available online at https://www.mdpi.com/2072-6643/12/6/1855/s1.
