# Supplementary material for: Unique Genetic and Histological Signatures of Mouse Pericardial Adipose Tissue
Source: Nutrients. 2020 Jun 22;12(6):1855. doi: 10.3390/nu12061855 (PMC7353424; doi:10.3390/nu12061855)
Supplement: Supplementary file 1 [file nutrients-12-01855-s001.pdf]

Supplementary Figure 1.

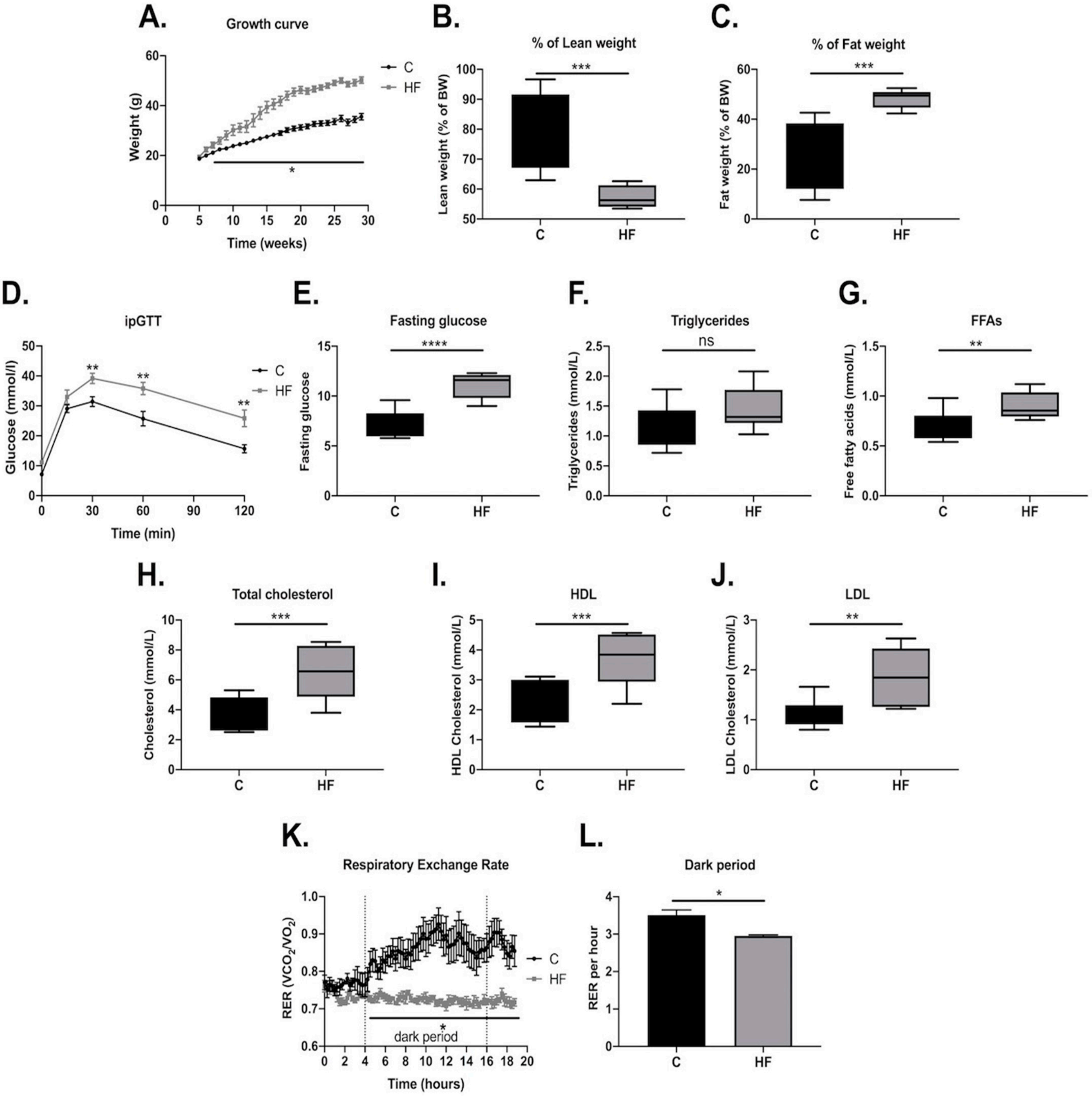

Supplementary Figure 1. Growth curve (A), % lean mass (B), % fat mass (C), intraperitoneal glucose tolerance test (IPGTT) (D), fasting glucose (E), plasma triglycerides (F), plasma free fatty acids (FFAs) (G), plasma total cholesterol (H), plasma HDL (I), plasma LDL (J), respiratory exchange ratio (K) during the dark period (L) in male mice fed either a chow (C) or high-fat (HF) diet. Data was analysed by an unpaired t-test. * $p < 0.05$, ** $p < 0.01$, *** $p < 0.001$ and **** $p < 0.0001$.

Supplementary Figure 2.

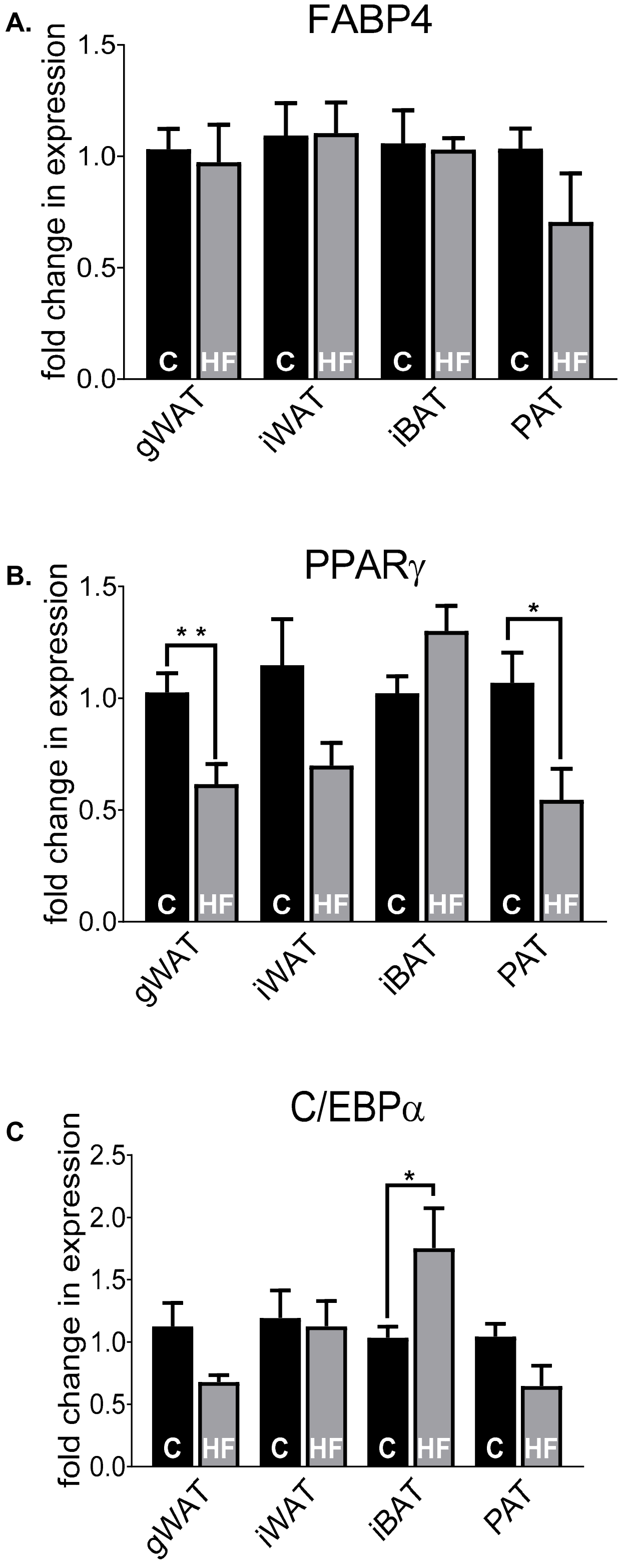

Supplementary Figure 2: FABP4 (A), PPARγ (B) and C/EBPα (C) fold change in mRNA expression in gWAT, iWAT, iBAT and PAT of chow (C) or high-fat (HF)-fed 30-week old male mice. All data is relative to the housekeeping gene PPIA and normalised to their respective control (C) within each depot. All data represents mean + s.e.m. Data was analysed by an unpaired t-test between C and HF of each depot. N = 6-12. * $p < 0.05$, ** $p < 0.01$, *** $p < 0.001$ and **** $p < 0.0001$.

Supplementary Figure 3.

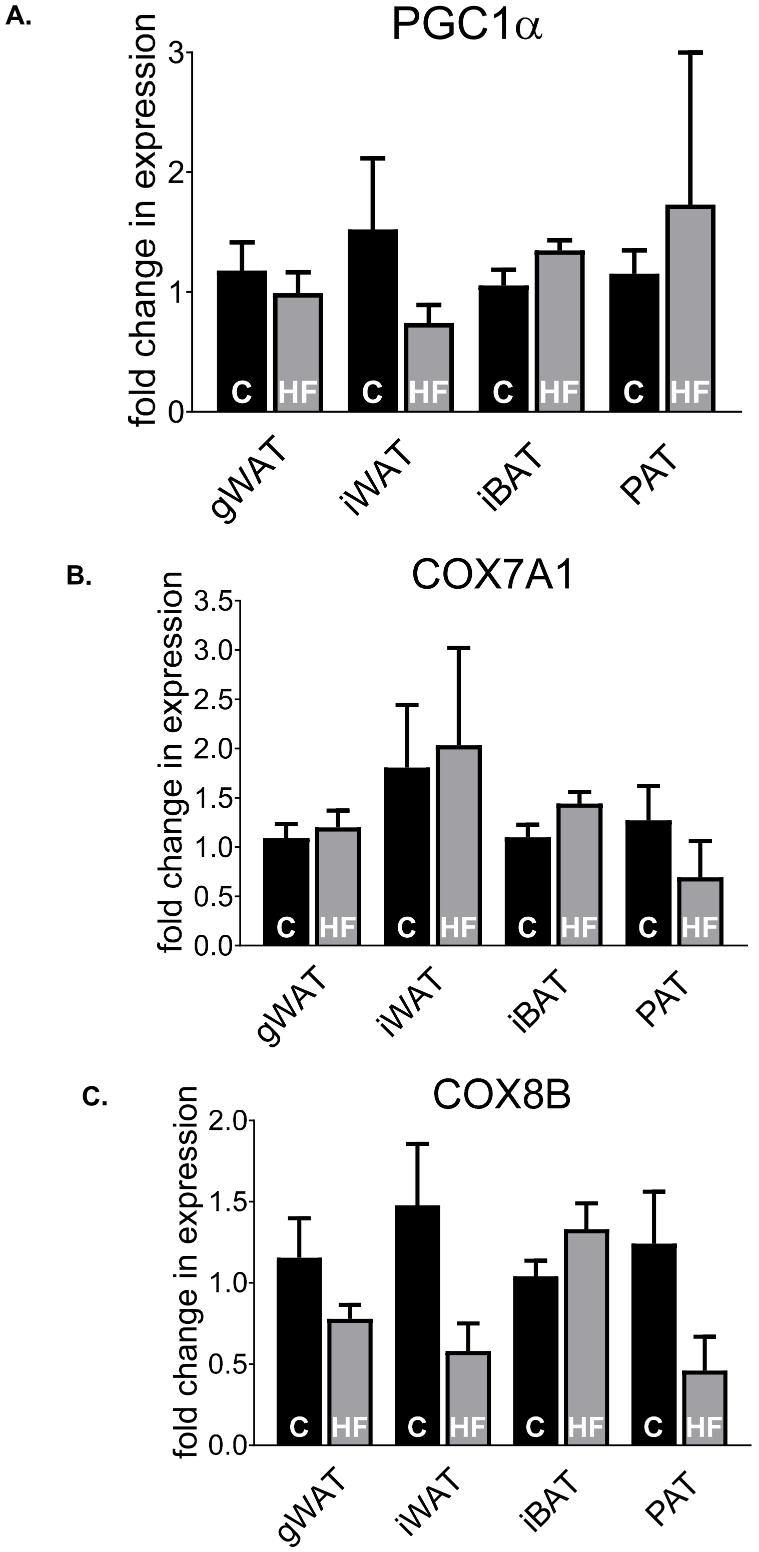

Supplementary Figure 3: PGC1α (A), COX7A1 (B) and COX8B (C) fold change in mRNA expression in gWAT, iWAT, iBAT and PAT of chow (C) or high-fat (HF)-fed 30-week old male mice. All data is relative to the housekeeping gene PPIA and normalised to their respective control (C) within each depot. All data represents mean + s.e.m. Data was analysed by an unpaired t-test between C and HF of each depot. N = 6-12. * $p < 0.05$, ** $p < 0.01$, *** $p < 0.001$ and **** $p < 0.0001$.

Supplementary Figure 4.

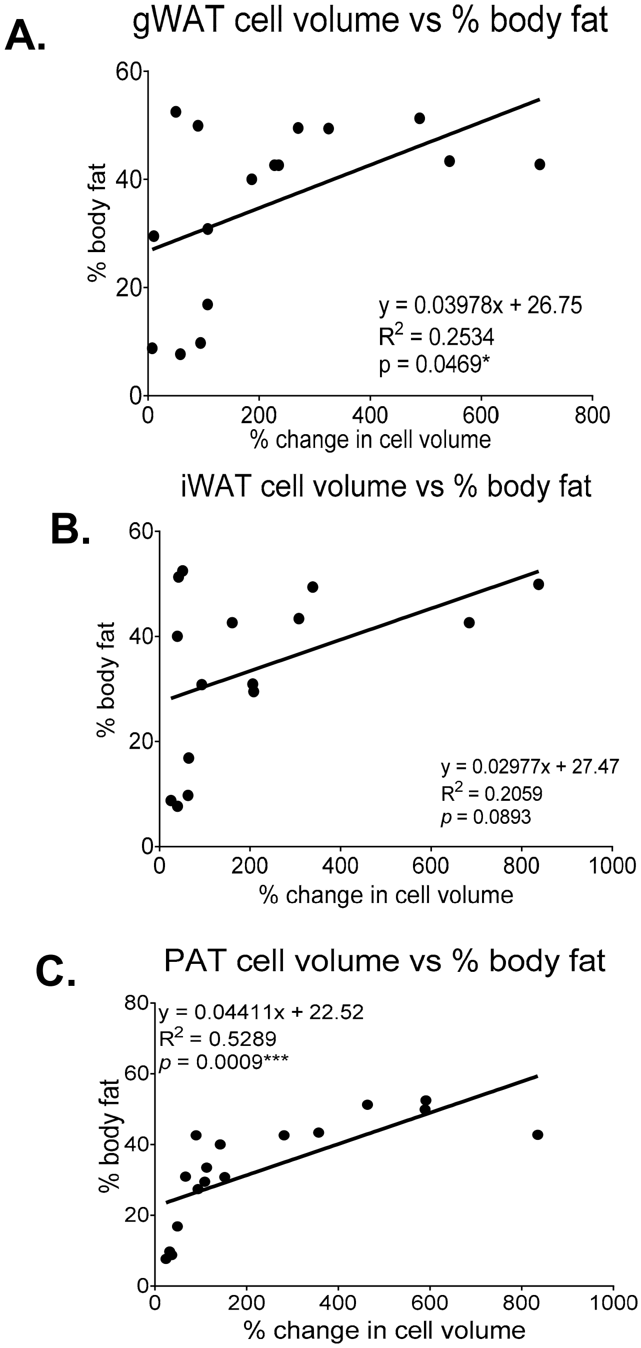

Supplementary Figure 4: The relationship between the percentage change in adipocyte cell volume (a.u.) in mice at 30-weeks old against the percentage body fat in mice at 26-weeks old in gWAT (A), iWAT (B) and PAT (C) of chow (C) and high-fat (HF)-fed mice. Percentage body fat was calculated by measuring the fat (g) and weight (g) of mice at 26-weeks old using Echo MRI and dividing fat by weight and multiplying by 100. All data represents individual data points plotted, with the line of best fit as determined by simple linear regression. Data was analysed by Pearon's correlation coefficient. N = 15-17. * $p < 0.05$, ** $p < 0.01$, *** $p < 0.001$ and **** $p < 0.0001$.

Supplementary Figure 5.

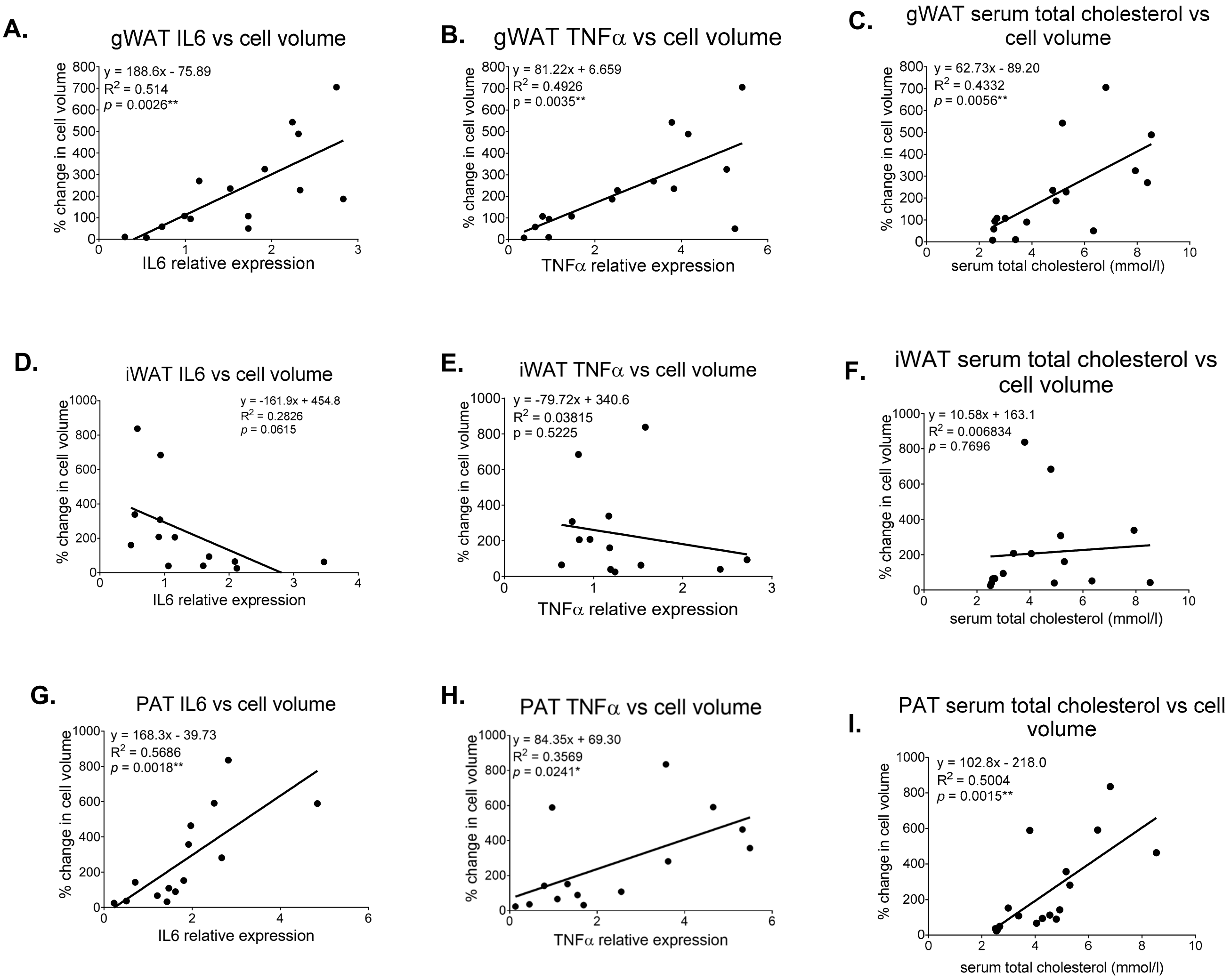

Supplementary Figure 5: The relationship between IL6, TNFα and serum total cholesterol against the percentage fold change in adipocyte volume (a.u.) in gWAT (A-C), iWAT (D-F) and PAT (G-I) of 30-week old chow (C) and high-fat (HF)-fed mice. All data represents individual data points plotted, with the line of best fit determined by simple linear regression. Data was analysed by Pearon's correlation coefficient. N = 13-17. * p < 0.05, ** p < 0.01, *** p < 0.001 and **** p < 0.0001.

Supplementary Figure 6

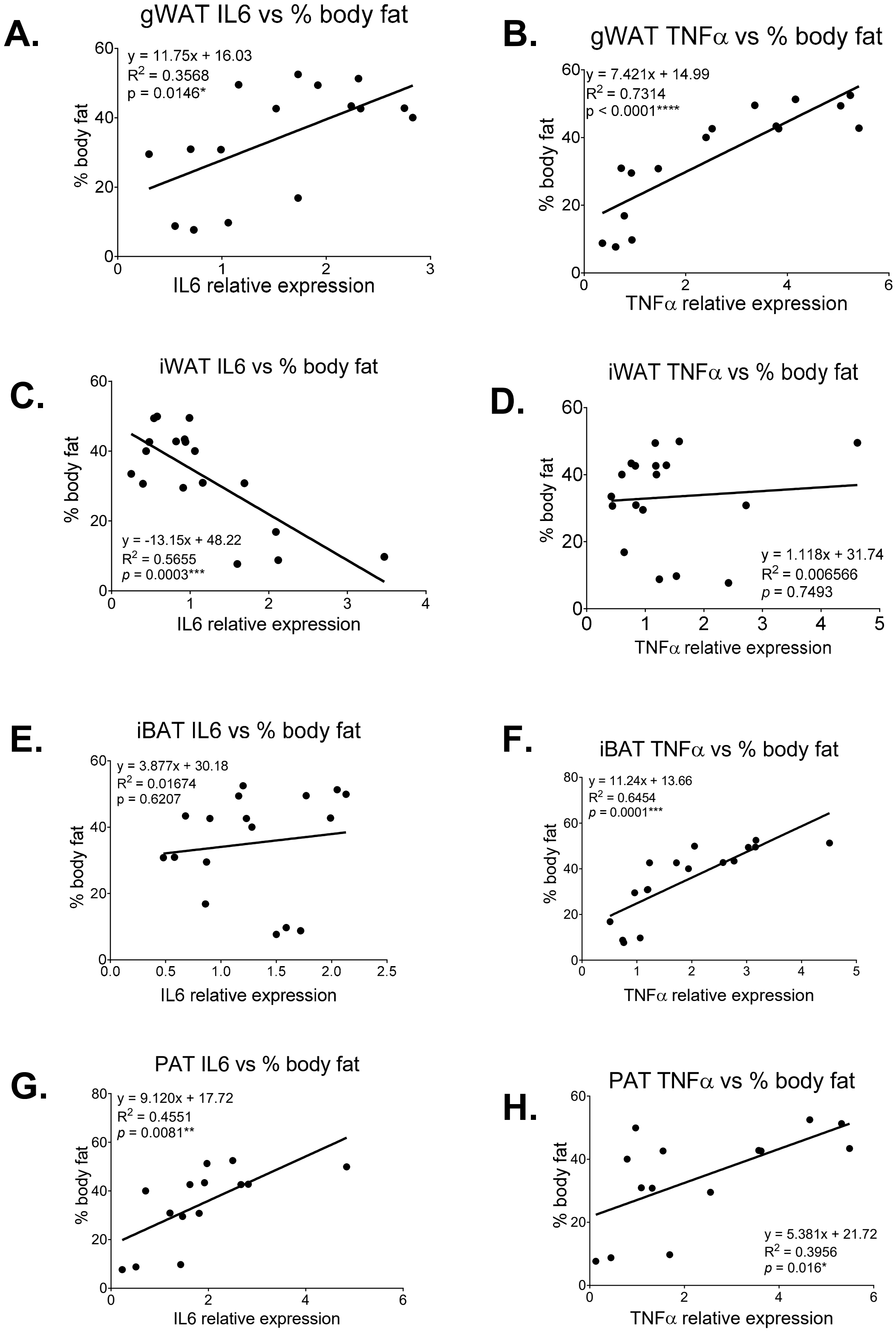

Supplementary Figure 6: The relationship between IL6 and TNFα of mice at 30-weeks old against the percentage body fat of mice at 26-weeks old in gWAT (A-B), iWAT (C-D), iBAT (E-F) and PAT (G-H) of chow (C) and high-fat (HF)-fed mice. Percentage body fat was calculated by measuring the fat (g) and weight (g) of mice at 26-weeks old using Echo MRI and dividing fat by weight and multiplying by 100.

All data represents individual data points plotted, with the line of best fit determined by simple linear regression. Data was analysed by Pearon's correlation coefficient. N = 14-17. * $p < 0.05$, ** $p < 0.01$, *** $p < 0.001$ and **** $p < 0.0001$.
